# Theatre Fires and Their Prevention

**Published:** 1903-01-03

**Authors:** 


### THEATRE FIRES AND THEIR PREVENTION.

Very few people are known to have been burned to death through fires in theatres, but very many have been crushed to death in the panic that always ensues when an alarm of fire is raised. For this reason the stringent regulations proposed by the London County Council may claim approval even from persons who do not think that the risk from fire renders them wholly necessary. If the audience at a theatre could only be convinced that they were in no danger when a fire broke out, they would in nine cases out of ten have plenty of time to escape without injury, even though in the end the place was burned to the ground. Therefore there must be ample assurance of safety, and this is the justification of precautions which may never be proved to have been necessary. Moreover, there has been of late years an increase in the number of theatre fires in spite of the fact that it is now more possible to prevent them than ever before. The employment of electric light instead of gas has done much to safeguard both actors and audience, but it is possible to do more. Wood, ropes, canvas, even gauze and muslin can now be rendered practically fireproof, and if managers were able to announce that neither in the construction, fittings, nor dresses employed in their theatres was there anything inflammable, they would go a long way to prevent the fatal panics that so often mark even a false alarm of fire. If the proprietors of theatres are willing to do this of their own accord, so much the better, and they would deserve to reap their reward in the greater popularity of their houses; but if they will not do so, it is certainly in the public interest that they should be compelled to make assurance doubly sure.

